# The Role of Sirtuin 1 (SIRT1) in Neurodegeneration

**DOI:** 10.7759/cureus.40463

**Published:** 2023-06-15

**Authors:** Daniel I Razick, Muzammil Akhtar, Jimmy Wen, Meraj Alam, Nabeal Dean, Muhammad Karabala, Ubaid Ansari, Zaid Ansari, Ethan Tabaie, Shakeel Siddiqui

**Affiliations:** 1 Surgery, California Northstate University College of Medicine, Elk Grove, USA; 2 Physical Medicine and Rehabilitation, California Northstate University College of Medicine, Elk Grove, USA; 3 Internal Medicine, California Northstate University College of Medicine, Elk Grove, USA; 4 Internal Medicine, University of California Berkeley, Berkeley, USA; 5 Neurosurgery, California Northstate University College of Medicine, Elk Grove, USA; 6 Anesthesiology, OrthoMed Staffing Anesthesiology Group, Dallas, USA

**Keywords:** alzheimer's disease, neurological diseases, histone deacetylase (hdac), neurodegeneration, sirtuin 1

## Abstract

Sirtuins (SIRT) are a class of histone deacetylases that regulate important metabolic pathways and play a role in several disease processes. Of the seven mammalian homologs currently identified, sirtuin 1 (SIRT1) is the best understood and most studied. It has been associated with several neurodegenerative diseases and cancers. As such, it has been further investigated as a therapeutic target in the treatment of disorders such as Parkinson’s disease (PD), Huntington’s disease (HD), and Alzheimer’s disease (AD). SIRT1 deacetylates histones such as H1 lysine 26, H3 lysine 9, H3 lysine 56, and H4 lysine 16 to regulate chromatin remodeling and gene transcription. The homolog has also been observed to express contradictory responses to tumor suppression and tumor promotion. Studies have shown that SIRT1 may have anti-inflammatory properties by inhibiting the effects of NF-κB, as well as stimulating upregulation of autophagy. The SIRT1 activators resveratrol and cilostazol have been shown to improve Alzheimer’s Disease Assessment Scale-Cognitive Subscale (ADAS-Cog) scores in AD patients. In this review, we aim to explore the various roles of SIRT1 with regard to neuroprotection and neurodegeneration.

## Introduction and background

Sirtuins (SIRT) are a family of NAD(+)-dependent class III histone deacetylases [1]. There are currently seven mammalian sirtuin homologs identified, from sirtuin 1 (SIRT1) to SIRT7, which regulate important metabolic pathways and vary greatly in their functions and locations [2]. SIRTs differ in length and sequence in their C- and N-terminal domains and thus localize differently. SIRT1 and SIRT2 localize in the nucleus and cytoplasm, while SIRT3-SIRT5 localize in the mitochondria [3,4]. They are best characterized by NAD(+)-dependent lysine deacetylation but have also been shown to remove other acyl groups such as succinyl, malonyl, and long-chain fatty acyl groups [5-8]. The enzymes are involved in several biological processes, including cell survival, proliferation, aging, longevity, senescence, apoptosis, DNA repair, and caloric restriction [9-11]. Recently, sirtuins have been considered potential targets for the treatment of a plethora of pathologies, including neurodegenerative, neoplastic, and cardiovascular diseases. Modulators of these enzymes have been of particular interest as they have been shown to have the potential for treating disorders such as type II diabetes, rheumatoid arthritis, cancer, and other aging-related diseases [12].

SIRT1 was the first sirtuin identified, which functions solely as a deacetylase and localizes in the nucleus and cytoplasm. This homolog is the best understood and most studied of the seven. It has been associated with neurodegenerative diseases and the following cancers: acute myeloid leukemia (AML), melanoma, glioma, lung adenocarcinoma, colon, prostate, ovarian, and breast [13]. With respect to Alzheimer’s disease (AD), SIRT1 activation via resveratrol has been found to inhibit NF-κB and diminish amyloid-β’s (Aβ) neurotoxic effect in microglia [14]. SIRT1 activity has also demonstrated a neuroprotective role in slowing neurodegenerative disease progression in pathologies such as Parkinson’s disease (PD) and amyotrophic lateral sclerosis (ALS) by upregulating autophagy [15]. SIRT1 has also been shown to be a promising therapeutic target for inhibiting p53 involvement in neurodegenerative diseases [16].

SIRT2 is also a deacetylase and localizes to the nucleus and cytoplasm. It has been associated with neurodegenerative diseases as well as gliomas. SIRT3 is a deacetylase that localizes to the mitochondria and has been associated with neurodegenerative diseases and the following cancers: B-cell chronic lymphocytic leukemia (CLL), mantle cell lymphoma, breast cancer, and gastric cancer. SIRT4 localizes to the mitochondria, functions as an ADP-ribosyltransferase, and is associated with breast and colorectal cancer. SIRT5 localizes to the mitochondria, acts as a malonyl, succinyl, and glutaryl deacetylase, and is associated with non-small cell lung carcinoma, pancreatic cancer, and breast cancer. SIRT6 localizes to the nucleus and is the only subtype that acts as a deacetylase, ADP-ribosyltransferase, and long-chain fatty acyl deacylase. SIRT7 localizes to the nucleus, functions as a deacetylase, and is associated with cancers of the liver, testis, spleen, thyroid, and breast [17].

Given the complex nature of the sirtuin family, we review the current literature and discuss the roles of SIRT1 with regard to neuroprotection and neurodegeneration.

## Review

SIRT1's mechanism of action

The sirtuin family is highly conserved throughout bacteria, archaea, and eukarya, suggesting the major role these protein-modifying enzymes serve. SIRTs are a family of class III NAD+-dependent histone deacetylases (HDACs). SIRTs are expressed ubiquitously and play essential roles as important regulators in a multitude of cellular processes such as DNA transcription, cell cycle progression, inflammation, and cell survival. Each SIRT possesses a conserved catalytic domain, NAD+ binding domains, distinct N and C terminals, and various substrate specificities, reflecting the functional differences between each SIRT [18]. The human SIRT1 gene is found on chromosome 10q22.1 with nine exons and eight introns, which produces a 747-amino acid residue protein. It is made up of the conserved catalytic core and the nuclear localization signal KRKKRK, identifying it as a nuclear protein. The N and C terminals for SIRT1 are among the largest in the SIRT family and are associated with increased SIRT1 catalytic activity [19]. The conserved catalytic core contains two NAD+ binding domains: a major Rossman fold and a minor domain composed of a helical module and a Zn2+ binding module [20,21]. 

SIRT1, like others in its family of class III HDACs, counteracts the actions of acetyl transferases by removing acetyl groups from histone and non-histone proteins. The catalytic reaction begins via the binding of the acetylated protein residue and NAD+ between these two domains. In this deacetylation reaction, the acetyl group is transferred onto the ribose component of NAD+, forming nicotinamide (NAM) and 2’-O-acetyl-ADP-ribose or 1’-O-acetyl-ADP-ribose and 3’-O-acetyl-ADP-ribose [22]. SIRT1 activity is regulated directly by cellular NAD+ levels, inhibition from its end product NAM, SIRT1-binding proteins, and post-translational modifications including ubiquitination, sumoylation, phosphorylation, glycosylation, nitrosylation, and glutathionylation. SIRT1 transcription is regulated by DNA methylation and a variety of transcription factors and cofactors, including p53, HiC1, E2F1, FoxO3a, and c-Myc [23,24]. SIRT1 expression also varies depending on different states of inflammation. It regulates inflammation through histone deacetylation of inflammatory cytokines and signaling pathways like NF-kB, HIF1a, AP-1, and P38MAPK [23]. 

SIRT1 deacetylates histones such as H1 lysine 26, H3 lysine 9, H3 lysine 56, and H4 lysine 16 to regulate chromatin remodeling and gene transcription. In the cytoplasm, SIRT1 deacetylates acetyl-CoA synthetase and cortactin, thus regulating fatty acid synthesis and cytoskeleton changes during cell migration, respectively [25,26]. SIRT1 is also controversial regarding its regulation of peroxisome proliferator-activated receptor gamma coactivator-1a (PGC-1a) and involvement in metabolism, mitochondrial biogenesis, and turnover [27]. The current understanding is that SIRT1 exerts an effect on mitochondrial function; however, the degree of influence varies depending on physiological context or cell type. In the mitochondria, SIRT1 and PGC-1a are associated with mtDNA and mitochondrial transcription factor A (TFAM). Its role in regulating PGC-1a underlies the potential of SIRT1 in affecting healthspan and longevity [24]. Imperatore et al. demonstrated the ability of SIRT1 to increase macrophage self-renewal and proliferative capacity both in vivo and in vitro [28]. SIRT1 inhibition has also been shown to negatively regulate E2F1, which is involved in G1/S transition and cell cycle progression, as well as the Myc and FoxO stress responses [29].

SIRT1 has been observed to deacetylate DNA damage repair enzymes such as WRN, APE1, XPA, and XPC and suppress tumor progression via inhibition of NF-kB [30]. Additionally, SIRT1 can promote tumor development through the deacetylation of tumor suppressors like HIC1, p53, p73, FoxO, Rb, and E2F1 [31]. Interestingly, nuclear receptors, cofactors, and oncogenes such as PPARy, androgen receptors (AR), estrogen receptors (ER), HIF-1a, AMP-activated protein kinase (AMPK), c-Myc, and N-Myc can be positively or negatively regulated by SIRT1. The mechanisms that SIRT1 exhibits in pathologies are complex due to the display of both tumor suppression and tumor-promoting features, as well as an observed dose-dependent effect on different cellular processes [32,33]. Given the contradictory role that SIRT1 demonstrates with regard to tumorigenesis, caution must be taken when activating the enzyme for therapeutic purposes, as tumor formation instead of suppression could result.

SIRT1 inhibition of NF-κB in neurodegenerative diseases

It is known that the transcription factor NF-κB is involved in promoting an inflammatory response, with p65 being an important subunit of NF-κB that is activated in chronic diseases such as neurodegenerative pathologies. The p65 subunit has seven acetylation sites on different lysine residues that, when acetylated, activate NF-κB thus leading to an inflammatory state [34]. In AD, there is a buildup of Aβ peptides that are presumed to cause microglial toxicity, leading to the neurodegenerative aspect of the disease. The buildup of Aβ peptides has experimentally been shown to be associated with activation of the NF-κB inflammatory pathway in surrounding glia via acetylation of its p65 subunit; this plays a major role in Aβ-dependent neurodegeneration in AD. The introduction of resveratrol, a SIRT1 activator, resulted in inhibition of NF-κB via deacteylation of p65 by SIRT1, thus diminishing Aβ’s effect of neurotoxicity in microglia [35]. Furthermore, higher serum levels of calcitriol, an active metabolite of vitamin D, have been experimentally shown to reduce the severity of PD symptoms by upregulating transcription of the SIRT1 gene, which encodes the SIRT1 protein. As SIRT1 levels increased, NF-κB levels decreased, suggesting an increase in SIRT1 deacetylase activity of the p65 subunit of NF-κB [36]. Further experiments show that PD also causes neuroinflammation by increasing levels of nitric oxide, which can modify SIRT1 by S-nitrosylation, thus inhibiting its deacetylase activity; at the same time, increased acetylation of the p65 subunit of NF-κB is observed [37]. These findings suggest that SIRT1 and sirtuin-activating compounds such as resveratrol and calcitriol may have a neuroprotective effect in AD, PD, and related neurodegenerative conditions by inhibiting the inflammatory effect of NF-κB.

Neuroprotective role of SIRT1/AMPK in upregulating autophagy

Neurodegenerative diseases such as PD and ALS have common characteristic features of the accumulation of irregular proteins within neurons, leading to the formation of inclusion bodies and the accumulation of dysfunctional mitochondria. This leads to dysfunctional autophagy, which promotes the aggressive nature of neurodegenerative diseases. While the toxic role of the inclusion bodies is not completely understood, they are heavily correlated with symptoms of various neurodegenerative diseases [38,39]. Furthermore, dysfunctional autophagy in oligodendrocytes has been shown to cause abnormal levels of myelination, which are consistent with the findings in neurodegenerative diseases such as AD [40]. SIRT1/AMPK activity has been shown to play a key role in autophagy by inducing mitochondrial fragmentation, which serves a neuroprotective role in slowing neurodegenerative disease progression [15]. Experiments have shown that activation of AMPK increases the intracellular NAD+/NADH ratio, leading to enhanced activity of SIRT1 [41]. SIRT1 can deacetylate and activate LKB1 kinase, which enhances its phosphorylation of AMPK, leading to a downstream increase in AMPK activity [42]. This suggests a positive feedback loop between SIRT1 and AMPK, which can potentiate the downstream neuroprotective effects of AMPK. A common feature of PD is impaired mitochondrial autophagy (mitophagy). A compound known as baicalein has been shown to have neuroprotective effects in PD rat models through the induction of the SIRT1/AMPK pathway. The addition of baicalein increased the activation of AMPK, which stimulated mitophagy, while the knockdown of SIRT1 in addition to baicalein annulled the autophagy activity. Further studies concluded that baicalein prevented neurotoxicity associated with PD by stimulating mitophagy via the SIRT1/AMPK axis, highlighting it as a therapeutic target in neurodegenerative diseases associated with impaired autophagy [43]. 

Huntington’s disease (HD) is associated with aggregates of mutant huntingtin (mHtt), which interfere with insulin signaling in neurons due to the accumulation of dysfunctional mitochondria. Stimulation of SIRT1/AMPK in HD-induced neuron models resulted in the upregulation of mitophagy, leading to a downstream effect of preservation of mitochondrial function indicated through the restoration of normal insulin signaling [44]. The SIRT1/AMPK axis is increasingly being recognized by researchers as a potential therapeutic target in neurodegenerative diseases where autophagy and mitophagy are impaired.

SIRT1 increases p53 deacetylation, decreasing the rate of neurodegeneration

Exposure of cells to various genotoxic stressors induces increased activity and amount of the tumor suppressor p53, leading to enhancement of its inhibition of cell growth, induction of apoptosis, and overall stability. Higher levels of p53 are commonly associated with the neurodegenerative aspects of diseases such as AD and PD [12]. Cell stress induces phosphorylation of p53 by Cdk5/p25 at various serine residues, thus inhibiting the degradation of p53, which translates to its accumulation. Further phosphorylation facilitates the binding of the cyclic AMP response element binding-binding protein (CREB-binding protein, or CBP), which functions to acetylate p53 at various lysine residues, resulting in enhanced p53 activity [45]. Induction of Cdk5/p25 in mice resulted in enhanced levels of Aβ peptide accumulation in the brain, which led to the conclusion that Aβ peptide levels were directly correlated with levels of Cdk5/p25. Increased levels of Cdk5/p25 were also shown to cause increased levels of active p53. These findings were consistent with the pathogenesis of AD [46]. The introduction of resveratrol, a SIRT1 activator, into the hippocampus of Cdk/p25 transgenic mice resulted in increased deacetylation and a decrease in the overall amount of p53. Increased SIRT1 also led to inhibition of the phosphorylation activity of Cdk/p25, leading to further inhibition of p53 activity. Overall SIRT1 activity led to a decreased rate of p53-induced cell death and eventual neurodegeneration seen in diseases such as AD and ALS [47]. Similarly, in PD models, decreased SIRT1 expression via induction of a specific miRNA resulted in increased p53 activity, which led to higher levels of cell death. This is consistent with the neurodegeneration seen in PD, and this study further highlighted the neuroprotective role of SIRT1 in decreasing neurodegeneration [48]. These studies again demonstrate SIRT1 as a promising therapeutic target to inhibit p53 involvement in neurodegenerative diseases. Along with resveratrol, other activators of SIRT1, such as olmesartan, show promising therapeutic results [16].

SIRT1's neuroprotective role in neurodegenerative diseases

In addition to its role in the hypothalamus, SIRT1 has been found to provide neuroprotection against neurological dysfunction [15]. The underlying importance of SIRT1 in protecting against neurodegenerative disease may be related to its regulation. AD is a neurodegenerative disorder characterized by the formation of neuritic senile plaques (NSPs) as well as neurofibrillary tangles (NFTs). These both contribute to neuronal death due to toxicity [49]. Recent studies suggest that the activity of SIRT1 may be involved in the interference of processes that produce both NSPs and NFTs [50]. Studies show that calorie restriction causes the activation of SIRT1, which then promotes AD neuroprotection by changing transcription factor activity [51]. The findings suggest that SIRT1 could potentially be a candidate for AD therapies, and with further investigation into its role in AD protection, targets for treating and preventing the disease may be provided.

HD is a hereditary neurodegenerative disorder that causes involuntary and irregular movements in the head, neck, face, and/or limbs. It is acquired via autosomal dominant inheritance through mutations in the CAG trinucleotide repeats in the huntingtin gene located on chromosome 4 [52]. Huntingtin plays a role within the central nervous system (CNS), but its exact function is not fully understood. Having over 40 or more CAG repeats results in the almost certain development of disease [53]. The neuroprotective effects of SIRT1 have shown promising results in mice models with HD. While its role in neuroprotection is still controversial, it has the potential to be utilized as a treatment option due to the lack of disease-modifying therapies for HD [54]. Brain-derived neurotrophic factor (BDNF) is a neurotrophic factor that plays an important role in neuronal survival and growth and serves as a neurotransmitter modulator [55]. Reduced transcription of BDNF has been indicated in the pathogenesis of HD, resulting in neural degeneration and dysfunction [56]. SIRT1 has been shown to preserve BDNF levels through transactivation at a promoter region for BDNF that is also regulated by the CREB transcription factor. Along with CREB activation, SIRT1 has also been shown to interact with the transducer of regulated CREB activity 1 (TORC1), which is an enhancer of CREB function and is involved in SIRT1-mediated regulation of BDNF transcription [57]. SIRT1 deacetylates TORC1 from specific lysine residues, which promote CREB and TORC1 interaction and ultimately promote transcription of BDNF [54].

Drugs that target SIRT1 and their use in AD treatment

Several pharmacological agents have been studied with regard to their effects on SIRT1. However, resveratrol and cilostazol are two SIRT1 activators that have been explored in depth. Resveratrol is a phytoalexin occurring mainly in grapevine species and other fruits. Given its antioxidant, anti-inflammatory, and neuroprotective activities, it has garnered much attention with respect to AD, PD, and HD treatment [14]. AD treatment has been of particular interest as there is evidence that resveratrol can decrease the aggregation of Aβ peptides in the hippocampus, prevent hippocampal damage, and promote neurogenesis [58]. Activation of SIRT1 by resveratrol has also been found to prevent Aβ-induced microglial death, which contributes to improved cognitive function [35].

Fang et al. conducted a clinical trial whereby patients with AD were given either donepezil (control) or a combination of donepezil and supplemental resveratrol. The results showed that patients given the combination therapy had higher mini-mental state examination (MMSE) scores and lower Alzheimer’s Disease Assessment Scale-Cognitive Subscale (ADAS-Cog) scores. The findings indicated that patients in the experimental group had improved cognitive function, self-care ability, and independence [59]. However, the subset of patients used in the study was from a small geographic region, and long-term results are still largely unknown. Therefore, greater patient diversity and long-term follow-up studies are needed to confirm the initial findings.

Moussa et al. performed a retrospective analysis on AD patients treated with oral resveratrol or a placebo for 52 weeks. The group found that patients treated with a placebo had a two-fold greater decline in MMSE scores, while those treated with resveratrol had a significant decrease in metalloproteinase-9 (MMP9) levels in the CSF. The decrease in MMP9 levels suggests that resveratrol may reduce CNS permeability, limiting the infiltration of leukocytes and other inflammatory agents into the brain. This study is limited by a small patient population with only 119 participants, and it was also mentioned that no significant difference was noted at baseline or at 52 weeks with regards to MMSE scores in the experimental group [60]. Given the population size and short-term data, further investigation is needed to determine the biomarker changes associated with resveratrol.

Frozza et al. compared the effects of free resveratrol with those of resveratrol-loaded lipid-core nanocapsule treatment against intracerebroventricular injection of Aβ1-42 in rats [61]. The findings showed higher concentrations of resveratrol nanoparticles in the brain than free resveratrol, resulting in increased bioavailability and possible neuroprotective effects. This study, in particular, highlights the potential beneficial effects of nano-drug delivery in the treatment of AD; however, it remains to be seen whether this holds true in human subjects as well.

Classified as a phosphodiesterase type III (PDE3) inhibitor, cilostazol is another SIRT1 inducer that has been investigated as a therapeutic agent in the treatment of AD [62]. Lee et al. sought to determine the efficacy of cilostazol administration in AD patients with white matter lesions. Participants were given either donepezil alone (control) or donepezil with cilostazol. The results showed that participants in the experimental group had significantly greater preservation of regional glucose metabolism in the left inferior frontal gyrus compared to those in the control group [63]. This increase in glucose metabolism was positively correlated with an improvement in participant ADAS-Cog scores. This study is limited by a small patient population of 36 and a short investigation period of 24 weeks. Long-term follow-up studies with a greater number of patients are needed to confirm the initial findings. Lee et al. set out to determine whether increased levels of cilostazol and consequent induction of SIRT1 would reduce Aβ peptide and APP-CTFβ levels in neuronal cells. After extensive investigation, the group found that cilostazol indeed increased Aβ clearance and increased cell viability by upregulating autophagy via activation of SIRT1 [64]. 

## Conclusions

The sirtuin family consists of NAD(+)-dependent class III histone deacetylases, which play an integral role in metabolic pathway regulation, DNA transcription, and cell cycle regulation. Sirtuins have been linked to several neurodegenerative pathologies and malignancies. SIRT1 has shown the potential to provide protection against neurodegenerative pathologies that involve abnormal protein accumulation, such as AD, PD, and ALS. SIRT1’s ability to inhibit NF-κB via deacteylation of p65 allows for mitigation of the inflammatory response, which can provide therapeutic benefit to patients suffering from these neurodegenerative disorders. In addition, SIRT1 promotes the phosphorylation of AMPK, which increases AMPK activity. This can provide a therapeutic effect through increased regulation of autophagy via mitochondrial fragmentation, which has been shown to slow neurodegenerative disease progression in PD and HD. In addition, SIRT1 has been shown to downregulate p53 activity, decreasing the amount of p53-induced cell death and helping to mitigate the effects of diseases like AD and ALS. The therapeutic effects of SIRT1 show promising potential for SIRT1 activators like resveratrol and cilostazol. These pharmacologic agents can be used to induce the effects of SIRT1’s anti-inflammatory and regulatory effects to decrease the rate of neurodegeneration. However, given the role of SIRT1 in tumor formation, caution must be taken when investigating therapeutic agents that activate the enzyme. While SIRT1 has been explored in depth, future research is prompted regarding its roles in other disease processes, such as cancer.
